# Impact of a Medical–Government Conflict on Healthcare Workers’ Mental Health in a Single Tertiary Hospital

**DOI:** 10.3390/jcm14238580

**Published:** 2025-12-03

**Authors:** Yechan Kyung

**Affiliations:** Department of Pediatrics, Samsung Changwon Hospital, Sungkyunkwan University School of Medicine, 51353 Changwon, Republic of Korea; drchany@naver.com

**Keywords:** physicians, nurses, occupational stress, anxiety, depression, COVID-19 pandemic

## Abstract

**Background/Objectives:** A medical–government conflict in South Korea in 2024 led to the collective resignation of resident physicians, causing severe workforce shortages in tertiary hospitals. This study aimed to investigate temporal changes in stress, anxiety, and depression among healthcare workers during this conflict following the COVID-19 pandemic. **Methods:** This retrospective study analyzed data from 56,137 adults who completed validated questionnaires on stress (KNHANES), anxiety (Clinically Useful Anxiety Outcome Scale, CUXOS), and depression (Center for Epidemiologic Studies Depression Scale, CES-D) between January 2019 and February 2025 at Samsung Changwon Hospital. Temporal trends were assessed using linear mixed-effects models adjusted for demographic variables. **Results:** Among attending physicians, stress increased by 1.44 points in the post-COVID period (*p* < 0.001) and 1.17 points during the conflict (*p* = 0.006), while anxiety increased by 1.25 (*p* = 0.012) and 1.36 points (*p* = 0.013), respectively. The most vulnerable subgroups were women, those aged 30–40 years, and physicians with <5 years of service. Depression increased significantly among physicians in their 40s during the post-COVID period (*p* = 0.018). Nurses demonstrated a significant reduction in stress during the conflict (−0.91, *p* = 0.002), possibly reflecting a temporary decrease in direct clinical workload, whereas office workers showed continuous increases in depression (+1.66 post-COVID, *p* = 0.003; +1.74 conflict, *p* = 0.001). **Conclusions:** The psychological effects of systemic crises differed by occupation. Attending physicians, who bore the greatest clinical and organizational burden, experienced persistent psychological strain during the medical–government conflict following post-pandemic stress. Tailored, occupation-specific strategies are required to protect healthcare workers’ mental health during future systemic disruptions.

## 1. Introduction

The mental health of healthcare workers is a critical public health concern that directly affects patient safety and the quality of care. Burnout, defined as emotional exhaustion, depersonalization, and reduced professional accomplishment, is closely associated with patient safety incidents, decreased patient satisfaction, and deterioration of professionalism [1]. Previous studies have reported that nearly half of physicians experience burnout, which is linked to turnover, early retirement, depression, anxiety, and even suicide risk [2,3]. Consequently, burnout and mental health problems are increasingly recognized not merely as issues of individual vulnerability, but as structural challenges within healthcare systems [4].

Infectious disease pandemics have clearly illustrated these challenges. During the Middle East Respiratory Syndrome (MERS) outbreak in South Korea in 2015, healthcare workers experienced high levels of stress, depression, and burnout due to infection risk, social stigma, and excessive workload [5,6]. Similarly, during the Coronavirus disease 2019 (COVID-19) pandemic, multiple studies reported a dramatic increase in burnout, depression, and anxiety among healthcare workers [7,8,9,10,11]. Shortages of personnel, infection risks, and excessive workloads contributed to mental health deterioration that persisted well beyond the acute phase of the pandemic [12,13].

Crucially, the impact of such workload intensification and structural stress is not uniform across all healthcare workers. Previous research indicates that the relationship between occupational stress and mental health varies significantly by job role, with physicians, nurses, and administrative staff exhibiting distinct vulnerabilities to changes in work intensity and organizational climate [14,15,16,17,18]. For instance, while physicians may be more affected by decision-making burdens and extended duty hours, nurses often face heightened emotional labor and physical exhaustion. Therefore, understanding these role-specific differences is essential for accurately assessing the consequences of systemic healthcare disruptions.

A similar but unique situation unfolded in South Korea in 2023, when the government’s announcement of a large-scale expansion of the medical school quota triggered a nationwide policy dispute. In response to the policy, resident physicians collectively resigned starting in March 2024, resulting in workforce shortages and substantial operational strain in tertiary hospitals. Essential clinical tasks previously handled by residents, such as night shifts and inpatient management, were shifted to attending physicians leading to a substantial increase in workload and psychological burden. This disruption not only exacerbated stress, depression, and anxiety among medical staff but also posed risks to patient safety and hospital operations. Unlike pandemics, such a large-scale medical–government conflict is rare and understudied, and no prior studies have evaluated its impact on healthcare workers’ mental health.

Therefore, the present study aimed to assess the impact of the medical–government conflict on the mental health of healthcare workers. Using health examination data collected between January 2019 and February 2025, we evaluated temporal changes in stress, anxiety, and depression among physicians, nurses, and other healthcare staff. This single-center health screening cohort is particularly appropriate for longitudinal analysis because its routine, standardized assessments enable reliable tracking of mental health changes over time. By providing empirical evidence, this study seeks to clarify the mental health consequences of such systemic conflicts and to inform future institutional and policy interventions.

## 2. Materials and Methods

### 2.1. Study Design and Population

This study was conducted as a retrospective observational study. The study population included adults who underwent health examinations at the Health Screening Center of Samsung Changwon Hospital, Sungkyunkwan University, between January 2019 and February 2025, and who completed all three mental health questionnaires assessing stress, anxiety, and depression. In South Korea, all employees are required by national policy to undergo an annual general health examination; however, completing this examination at our hospital’s screening center is not mandatory. Therefore, the repeated assessments captured in this study reflect individuals who voluntarily chose to receive their examinations at this facility. Demographic and occupational information, including sex, age, educational level, occupation, length of service, and marital status, was collected for all participants. Individuals who did not respond to one or more questionnaire items, or who had missing data on key demographic variables such as sex, age, or occupation, were excluded from the analysis. Participants were classified as healthcare workers or non-healthcare workers, with healthcare workers further categorized into attending physicians, nurses, office workers, and other staff. The other staff included allied health professionals such as medical laboratory technologists, radiologic technologists, rehabilitation therapists, and pharmacists. Among attending physicians, subgroup analyses were performed according to sex, age, and length of service.

### 2.2. Period Classification

To evaluate temporal changes in mental health questionnaire scores, the study period was divided into four distinct phases: the pre-COVID period, the COVID period, the post-COVID period, and the medical–government conflict period. The pre-COVID period was defined as 1 January to 31 December 2019, when the healthcare environment in Korea remained unaffected by COVID-19. The COVID period was defined as from 1 January 2020, considering the date of the first confirmed case in Korea, through 31 May 2023, corresponding to the nationwide termination of outdoor mask mandates on 26 May 2023. The post-COVID period was defined as 1 June 2023 to 29 February 2024. This interval, following the cessation of major national public health mandates, was crucial for assessing the psychological state of healthcare workers after the acute phase of the pandemic and the subsequent structural relief from high infection anxiety and mandated workload, establishing a recovery baseline before the onset of the medical–government conflict. Finally, the medical–government conflict period was defined as 1 March 2024 to 28 February 2025, commencing with the widespread collective resignation of resident physicians across the nation, which resulted in severe workforce shortages and structural changes in tertiary hospital operations.

### 2.3. Measurement Tools

Stress levels were assessed using a 9-item questionnaire adopted from the Korea National Health and Nutrition Examination Survey (KNHANES) [19]. Each item was rated on a 5-point scale, yielding a total score ranging from 9 to 45, with higher scores indicating higher levels of perceived stress. Depression levels were measured using the Center for Epidemiologic Studies Depression Scale (CES-D) [20]. The CES-D consists of 20 items, each scored from 0 to 3, with a total score ranging from 0 to 60; higher scores indicate greater severity of depression, and scores of 16 or higher were considered indicative of possible clinical depression. Anxiety levels were evaluated using the Clinically Useful Anxiety Outcome Scale (CUXOS) [21]. The CUXOS comprises 20 items rated on a 5-point Likert scale (0–4), with a total score ranging from 0 to 80. Higher scores reflect greater severity of anxiety, which was categorized as mild (0–25), moderate (26–50), and severe (51–80). All questionnaires used in this study were validated Korean versions with established reliability and validity. The internal consistency of all instruments was high in this dataset, with Cronbach’s α values of 0.90 (KNHANES stress scale), 0.87 (CES-D), and 0.94 (CUXOS).

### 2.4. Statistical Analysis

Baseline characteristics of the study population were summarized as means with standard deviations for continuous variables and as frequencies with percentages for categorical variables. Group differences were assessed using one-way analysis of variance for continuous variables and Pearson’s chi-square test for categorical variables. Baseline characteristics were derived from the questionnaires completed at the first visit, whereas temporal changes in mental health questionnaire scores were analyzed by including all repeated responses from the same individual. Longitudinal changes over time were examined using linear mixed-effects models. The variables included in the models were defined as follows: the independent variable was the health-examination period; the dependent variables were stress, anxiety, and depression scores; and the covariates were age, sex, educational level, and marital status. Variables such as smoking, alcohol consumption, and obesity were excluded from the covariate set to avoid overadjustment bias, as these factors may act as mediators on the causal pathway [22,23,24]. To assess the potential influence of seasonality on mental health outcomes, we conducted a sensitivity analysis adjusting for calendar month. The period-specific estimates remained consistent in both direction and magnitude, indicating that seasonal effects did not materially affect the findings. The linear mixed-effects models included random intercepts for participant IDs to account for baseline heterogeneity and within-person correlation across repeated measurements. Intraclass correlation ranged from 0.71 to 0.94, indicating substantial within-person clustering of repeated mental health measures. Random slopes for time were not included to maintain model stability given the large sample size and irregular assessment intervals. Among attending physicians, analyses were additionally stratified by length of service, categorized as <5 years, 5–14 years, and ≥15 years. All statistical analyses were performed using Stata version 17 (StataCorp, College Station, TX, USA), and statistical significance was defined as a two-sided *p*-value of <0.05.

## 3. Results

Between January 2019 and February 2025, a total of 243,252 individuals underwent health examinations at the Health Screening Center of Samsung Changwon Hospital. Among them, 187,115 individuals were excluded due to missing responses in one or more of the three mental health questionnaires. The final analytic sample consisted of 56,137 participants who completed all assessments on stress, anxiety, and depression. These participants contributed a total of 171,195 health examination records, with an average of 3.05 ± 1.98 (mean ± SD) repeated assessments per individual during the study period. Of these, 54,122 were non-healthcare workers, 237 were attending physicians, 1113 were nurses, 234 were office workers, and 431 were other hospital staff (Table 1).

Overall, 73.8% of the participants were male and 26.2% were female. The sex distribution differed significantly by occupation: the proportion of males was highest among attending physicians (70.0%), whereas females predominated among nurses (93.3%) (*p* < 0.001). The mean age of the entire cohort was 40.0 years, with attending physicians being the oldest group (43.0 years) and nurses the youngest (33.2 years) (*p* < 0.001). By age distribution, physicians were most frequently in their 40s (32.9%), whereas nurses were most frequently in their 20s (44.4%) (*p* < 0.001). The mean length of service was 10.3 years overall, with the shortest among physicians (4.8 years) and the longest among office workers (11.7 years) (*p* < 0.001). The proportion with <5 years of service was highest among physicians (69.2%), whereas the proportion with ≥15 years was greatest among office workers (32.1%) (*p* < 0.001).

The mean stress score was 15.3 points overall, with the highest in office workers (16.3) and relatively lower in physicians (15.0) (*p* < 0.001). The mean anxiety score, assessed by the CUXOS, was 12.4 overall, 12.2 among nurses, and 10.9 among physicians, the latter being significantly lower than the overall mean (*p* = 0.002). Regarding anxiety severity, 85.3% of nurses and 92.8% of physicians were classified as having mild anxiety, whereas 13.8% of nurses and 6.3% of physicians reported moderate anxiety. The proportion with severe anxiety was <1% in both groups. The mean CES-D depression score was 8.4 overall. A score ≥16, indicating possible clinical depression, was observed in 10.7% of the total sample. This proportion was 9.3% among nurses, similar to the overall mean, but lowest among physicians at 6.3% (*p* = 0.004).

### 3.1. Temporal Changes in Mental Health Status by Occupation

**Figure 1:**
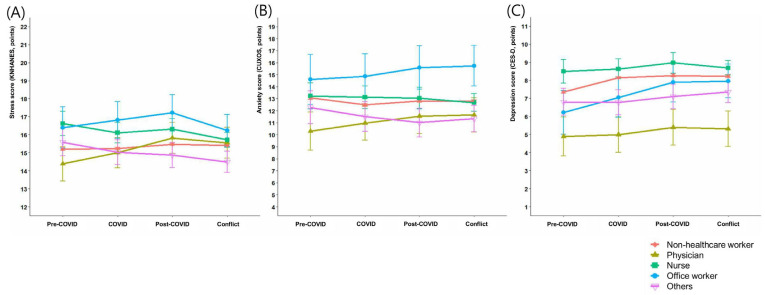
Trends in mental health questionnaire scores over time according to occupation. (**A**) Stress scores assessed using the KNHANES questionnaire, (**B**) anxiety scores assessed using the CUXOS, and (**C**) depression scores assessed using the CES-D. For physicians, both stress and anxiety scores significantly increased during the post-COVID and medical–government conflict periods compared with the pre-COVID period. In contrast, nurses showed a significant decrease in stress scores during the medical–government conflict period compared with the pre-COVID period.

Temporal changes by occupation were analyzed using linear mixed-effects models (Table 2, Figure 1). Among non-healthcare workers, stress scores increased significantly compared with the pre-COVID period, by 0.26 points during the post-COVID period and 0.20 points during the medical–government conflict period (both *p* < 0.001). In attending physicians, stress scores increased more markedly than in other occupational groups, by 1.44 points in the post-COVID period (*p* < 0.001) and 1.17 points during the conflict period (*p* = 0.006). In contrast, stress scores decreased significantly during the conflict period among nurses (−0.91, *p* = 0.002) and other staff (−1.09, *p* = 0.001). Anxiety scores consistently decreased across all periods among non-healthcare workers (all *p* < 0.001), whereas they increased among attending physicians by 1.25 points in the post-COVID period (*p* = 0.012) and 1.36 points during the conflict period (*p* = 0.013). Depression scores increased significantly across all periods among non-healthcare workers (all *p* < 0.001), with office workers showing the most pronounced increases, by 1.66 points in the post-COVID period (*p* = 0.003) and 1.74 points during the conflict period (*p* = 0.001). No significant changes in depression scores were observed among nurses or other staff.

### 3.2. Subgroup Analyses of Attending Physicians by Sex, Age, and Length of Service

**Figure 2:**
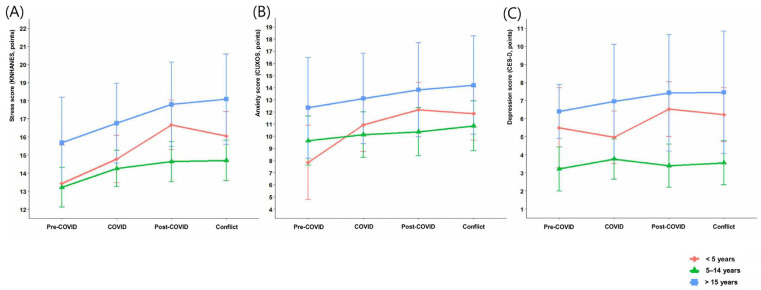
Trends in mental health questionnaire scores over time according to length of service among physicians. (**A**) Stress scores assessed using the KNHANES questionnaire, (**B**) anxiety scores assessed using the CUXOS, and (**C**) depression scores assessed using the CES-D. In physicians with less than 5 years of service, stress scores significantly increased during the post-COVID and medical–government conflict periods compared with the pre-COVID period, and anxiety scores significantly increased during the COVID, post-COVID, and medical–government conflict periods. In those with 5–14 years of service, stress scores significantly increased during the COVID, post-COVID, and medical–government conflict periods. Physicians with 15 or more years of service showed no statistically significant changes in mental health questionnaire scores.

Subgroup analyses were performed among attending physicians, who were expected to experience the greatest burden due to the collective withdrawal of residents, according to sex, age, and length of service. In sex-stratified analyses, stress scores in male physicians increased by 1.14 points in the post-COVID period (*p* = 0.015), whereas female physicians showed larger increases of 2.08 points in the post-COVID period (*p* = 0.008) and 1.73 points during the conflict period (*p* = 0.030). By age group, physicians in their 30s exhibited the greatest increases in stress, with 3.13 points in the post-COVID period (*p* = 0.007) and 3.18 points during the conflict period (*p* = 0.008). Physicians in their 40s also showed significant increases of 1.90 (*p* = 0.001) and 1.64 points (*p* = 0.006), respectively. Anxiety scores increased among male physicians in the post-COVID (1.42, *p* = 0.014) and conflict periods (1.45, *p* = 0.024), and in physicians in their 30s and 40s across both periods. Depression scores increased significantly only among physicians in their 40s, by 1.25 points during the post-COVID period (*p* = 0.018). By length of service, physicians with <5 years showed significant increases in stress scores during the post-COVID (3.25, *p* = 0.002) and conflict periods (2.62, *p* = 0.018), along with consistent increases in anxiety scores across all periods. Physicians with 5–14 years of service showed significant increases only in stress scores, whereas those with ≥15 years showed no significant changes in stress, anxiety, or depression (Table 3, Figure 2).

## 4. Discussion

This study represents the first large-scale longitudinal analysis to examine changes in mental health across different occupational groups of healthcare workers during two consecutive societal and policy crises: the COVID-19 pandemic and the subsequent medical–government conflict. In particular, we identified vulnerable subgroups among attending physicians, who carried the greatest burden in the absence of residents, according to sex, age, and length of service. Stress and anxiety increased significantly among physicians during both the post-COVID and conflict periods, with the most pronounced vulnerability observed in women, those in their 30s and 40s, and those with less than 5 years of service. Nurses, who initially reported high levels of stress and depression, showed a decrease in stress during the conflict period. Office workers exhibited a cumulative increase in depression during both the post-COVID and conflict periods. These findings suggest that both infectious disease crises and medical–government conflicts affect healthcare workers differently according to their occupational role, and that attending physicians showed persistent elevations in stress and anxiety from the COVID-19 period through the medical–government conflict.

To clarify whether these changes reflected occupation-specific effects rather than broader societal trends, we also examined temporal patterns in the non-healthcare worker reference group. This group showed only minimal fluctuations across the defined periods. Although these changes reached statistical significance due to the large sample size, the absolute differences were small, indicating a relatively stable mental health trajectory in the general population. This stability suggests that the substantial changes observed among attending physicians were more occupation-specific than attributable to broader societal trends.

These differences may be related to the redistribution of clinical duties and the reduction in outpatient services and surgeries following the withdrawal of residents. Among attending physicians, the absence of residents was linked to a concentration of night shifts and clinical workload, and this burden may have been particularly pronounced among female physicians, those in their 30s and 40s, and those with less than 5 years of service. These groups often occupy early-career positions in which clinical responsibility increases rapidly, while simultaneously facing greater challenges related to childcare and family obligations. Such structural and personal factors may help explain their heightened vulnerability during the conflict period [25,26,27]. In contrast, the decrease in stress observed among nurses may be explained by the fact that the conflict was centered on physicians and the government, while the reduction in clinical services may have been associated with a partial reduction in workload. Furthermore, previous studies have shown that physician–nurse conflict is strongly associated with nurses’ occupational stress; thus, the absence of residents may have temporarily alleviated such conflicts, contributing to reduced stress levels among nurses [28,29,30]. For office workers, the persistent increase in depression scores may reflect uncertainty about hospital operations and increased administrative burden. In addition, concurrent institutional changes and broader economic stressors during this period—such as inflation and rising interest rates—may have contributed to greater psychological vulnerability. Because office staff were less directly involved in the medical–government conflict, their mental health may have been influenced more by organizational instability and external economic pressures than by clinical workload.

Previous infectious disease crises have also demonstrated that excessive workload can have severe adverse effects on the mental health of healthcare workers. During the 2015 MERS outbreak in Korea, physicians reported extremely high levels of burnout, with 79.5% experiencing exhaustion and 65.5% depersonalization; additionally, working in a hospital where confirmed cases occurred was identified as a major factor, increasing the risk of depression more than fourfold [5,6]. Similarly, during the COVID-19 pandemic, the prevalence of depression, anxiety, stress, and burnout among healthcare workers was markedly higher than in the general population, with long working hours, uncertain clinical environments, and fear of infection identified as key contributors [7,31,32]. The observed increases in stress and anxiety among attending physicians during the post-COVID and conflict periods are consistent with these prior findings, indicating that the workload associated with pandemic response remained elevated and was related to adverse mental health patterns even during the subsequent medical–government conflict.

Although the increased stress and anxiety among attending physicians are consistent with findings from previous crises such as MERS and COVID-19, the pattern observed in this study differs in several important ways. Infectious disease outbreaks typically elevated distress across all frontline professions, whereas the medical–government conflict was associated with more uneven impact—showing worsening scores among physicians, decreasing stress among nurses, and increasing depression among office workers. This contrast suggests that internal structural crises may generate more role-specific psychological pressures than external infectious threats.

It has been well documented that the vulnerability of mental health differs according to the occupational group of healthcare workers. A large-scale survey in the United States reported high levels of burnout in both physicians and nurses, but identified differences in underlying causes and preferred interventions, highlighting the need for occupation-specific approaches [33]. More recently, a network analysis demonstrated that the structural associations between depression and burnout differed between physicians and nurses, suggesting that the manifestation of mental health problems may fundamentally vary by occupation [34]. Another study that included all hospital staff found that, in addition to physicians and nurses, administrative and other personnel also experienced mental health burdens after COVID-19, with distinct patterns of anxiety, depression, and stress reported across occupational groups [35]. Furthermore, one study showed that the mental health impact of the COVID-19 pandemic was particularly pronounced in frontline healthcare workers with long working hours and high exposure to infection risk [36]. Our findings are consistent with these prior reports, demonstrating that while attending physicians exhibited persistent deterioration in mental health across both the post-COVID and medical–government conflict periods, nurses showed a reduction in stress during the conflict period, and office workers experienced a cumulative increase in depression. These results underscore that even under the same crisis, the mental health impact may vary substantially depending on the occupational role and division of responsibilities.

Mental health problems among healthcare workers have profound implications for the healthcare system as a whole, being directly linked to patient safety, quality of care, and patient satisfaction [2]. Individual resilience or self-coping strategies may serve as partial protective factors; however, multiple studies have shown that their effects are limited under conditions of extreme workload and structural constraints such as those experienced during the COVID-19 pandemic [37,38,39]. Indeed, even among physicians with high resilience, a considerable proportion still reported burnout, indicating that personal effort alone is insufficient to counter these challenges [40]. A recent meta-analysis further demonstrated that the risk of burnout increases proportionally with higher levels of job stress and emphasized that organizational and policy-level interventions, such as adequate staffing, work hour adjustments, reduction in administrative burdens, and psychological support, are essential to mitigate these risks [41]. In this context, our finding that stress and anxiety among attending physicians persisted beyond the COVID-19 period into the medical–government conflict underscores the need for systemic and multifaceted strategies, rather than reliance solely on individual resilience, to safeguard the mental health of healthcare workers during crises.

In Supplementary categorical analyses applying established clinical thresholds—such as the CES-D cutoff of 16 points for possible depression and the CUXOS threshold for moderate anxiety—the odds of belonging to these clinical risk groups did not show significant changes across the defined periods in any occupational category (Tables S4-1 and S4-2). This contrast between statistically significant shifts in continuous scores and the absence of meaningful increases in clinically elevated cases suggests that the psychological impact observed in this health screening cohort was largely subclinical. These findings highlight the importance of distinguishing statistical significance from clinical relevance when interpreting mental health trends derived from screening-based populations.

This study has several notable strengths. First, it represents the first investigation in South Korea to analyze the mental health status of tertiary hospital healthcare workers before and after the 2023 medical–government conflict. Second, by sequentially comparing the pre-COVID, COVID, and medical–government conflict periods, this study allowed for the evaluation of how mental health deterioration observed during an infectious disease crisis relates to, or differs from, that observed during a large-scale policy-driven conflict. Third, by utilizing longitudinal data accumulated through periodic health examinations, this study assessed long-term trends in mental health across diverse occupational groups within a single hospital, rather than relying on a single cross-sectional assessment.

However, this study also has several limitations. First, as a retrospective study conducted in a single tertiary medical center, the possibility of selection bias cannot be excluded, and the generalizability of the findings may therefore be limited. In particular, the results may have been shaped by regional characteristics—such as patient volume patterns and the socioeconomic context of Changwon—as well as hospital-specific operational policies during the collective resignation period. As a result, the magnitude or pattern of the observed effects may not be fully generalizable to other tertiary hospitals or settings. Second, because mental health assessments were based on self-reported questionnaires rather than clinical interviews, recall bias may have occurred. Additionally, among attending physicians in this cohort, the proportion with less than 5 years of service was relatively high. This imbalance raises the possibility of residual confounding, as mental health patterns could differ systematically according to seniority, career stage, or generational factors. Third, the study lacked direct indicators of workload—such as working hours, night-shift burden, or patient volume—which limits the ability to determine whether the observed occupation-specific changes in stress and anxiety were truly driven by shifts in clinical responsibilities. Fourth, several key covariates—particularly education level and marital status—showed relatively high rate of missing data (28.7% and 28.4%, respectively). Although age and sex were complete, the incomplete availability of these sociodemographic variables raises the possibility of residual confounding, as the adjusted models may not have fully captured their influence on mental health outcomes.

## 5. Conclusions

In this longitudinal study spanning the COVID-19 pandemic and the medical–government conflict, attending physicians—especially women and early-career clinicians—showed persistent increases in stress and anxiety, whereas nurses and office workers exhibited different and more variable mental health patterns. These findings highlight that crises impose uneven psychological burdens across occupational groups and call for tailored support strategies rather than uniform interventions. Targeted measures such as structured peer support, workload redistribution, and organizational stabilization may help address the distinct needs identified. Although generalizability is limited by the single-center design and reliance on self-reported data, the study underscores the need for system-level, occupation-specific strategies to protect healthcare workers’ mental health during future disruptions and supports the value of further multicenter research.

## Figures and Tables

**Table 1:** Baseline characteristics of the study population.

	Total (N = 56,137)	Non-Healthcare Worker (N = 54,122)	Physician (N = 237)	Nurse (N = 1113)	Office Worker (N = 234)	Other Staff (N = 431)	*p*-Value
Sex							<0.001
Male	41,407 (73.76)	40,859 (75.49)	166 (70.04)	75 (6.74)	88 (37.61)	219 (50.81)	
Female	14,730 (26.24)	13,263 (24.51)	71 (29.96)	1038 (93.26)	146 (62.39)	212 (49.19)	
Age, mean ± SD	40.04 ± 12.36	40.21 ± 12.43	42.97 ± 8.87	33.18 ± 8.22	38.02 ± 8.77	36.66 ± 8.86	<0.001
20–29	13,344 (23.77)	12,683 (23.43)	3 (1.27)	494 (44.38)	43 (18.38)	121 (28.07)	<0.001
30–39	18,967 (33.79)	18,180 (33.59)	108 (45.57)	406 (36.48)	103 (44.02)	170 (39.44)	
40–49	11,459 (20.41)	11,086 (20.48)	78 (32.91)	144 (12.94)	60 (25.64)	91 (21.11)	
50–59	8144 (14.51)	7969 (14.72)	35 (14.77)	68 (6.11)	24 (10.26)	48 (11.14)	
≧60	4223 (7.52)	4204 (7.77)	13 (5.49)	1 (0.09)	4 (1.71)	1 (0.23)	
Length of service, mean ± SD	10.34 ± 8.44	10.54 ± 8.43	4.84 ± 6.38	7.64 ± 7.93	11.70 ± 9.53	9.28 ± 8.64	<0.001
<5 yr	7387 (13.16)	6392 (11.81)	164 (69.20)	588 (52.83)	76 (32.48)	167 (38.75)	<0.001
5–14 yr	9942 (17.71)	9285 (17.16)	49 (20.68)	359 (32.26)	83 (35.47)	166 (38.52)	
≧15 yr	6366 (11.34)	6003 (11.09)	24 (10.13)	166 (14.91)	75 (32.05)	98 (22.74)	
Education level							<0.001
High school or below	16,257 (28.96)	16,059 (29.67)	0 (0.00)	100 (8.98)	60 (25.64)	38 (8.82)	
College	21,247 (37.85)	19,700 (36.40)	76 (32.07)	950 (85.35)	155 (66.24)	366 (84.92)	
Graduate school or higher	2522 (4.49)	2252 (4.16)	161 (67.93)	63 (5.66)	19 (8.12)	27 (6.26)	
Work schedule							<0.001
Three-shifts	4921 (8.77)	4296 (7.94)	1 (0.42)	584 (52.47)	8 (3.42)	32 (7.42)	
Two-shifts	6912 (12.31)	6848 (12.65)	3 (1.27)	27 (2.43)	1 (0.43)	33 (7.66)	
Every-other-day shift	52 (0.09)	17 (0.03)	2 (0.84)	2 (0.18)	1 (0.43)	30 (6.96)	
Fixed night shift	57 (0.10)	32 (0.06)	8 (3.38)	5 (0.45)	0 (0.00)	12 (2.78)	
Other	752 (1.34)	540 (1.00)	77 (32.49)	64 (5.75)	2 (0.85)	69 (16.01)	
Marital status							<0.001
Never married	9782 (17.43)	8787 (16.24)	49 (20.68)	663 (59.57)	93 (39.74)	190 (44.08)	
Married	28,739 (51.19)	27,757 (51.29)	185 (78.06)	428 (38.45)	138 (58.97)	231 (53.60)	
Separated	105 (0.19)	103 (0.19)	0 (0.00)	1 (0.09)	0 (0.00)	1 (0.23)	
Divorced	997 (1.78)	970 (1.79)	2 (0.84)	16 (1.44)	2 (0.85)	7 (1.62)	
Widowed	575 (1.02)	566 (1.05)	1 (0.42)	5 (0.45)	1 (0.43)	2 (0.46)	
Mental health diagnosis							<0.001
Yes	2541 (4.53)	2519 (4.65)	6 (2.53)	7 (0.63)	5 (2.14)	4 (0.93)	
No	53,596 (95.47)	51,603 (95.35)	231 (97.47)	1106 (99.37)	229 (97.86)	427 (99.07)	
Stress score, mean ± SD	15.25 ± 6.50	15.23 ± 6.51	15.03 ± 5.86	15.95 ± 6.36	16.26 ± 6.48	14.90 ± 5.73	<0.001
Anxiety score, mean ± SD	12.37 ± 12.39	12.38 ± 12.42	10.89 ± 10.76	12.22 ± 11.64	14.34 ± 12.51	10.73 ± 11.00	0.002
0–25	47,213 (84.10)	45,478 (84.03)	220 (92.83)	949 (85.27)	186 (79.49)	380 (88.17)	0.001
26–50	8483 (15.11)	8217 (15.18)	15 (6.33)	154 (13.84)	47 (20.09)	50 (11.60)	
51–80	441 (0.79)	427 (0.79)	2 (0.84)	10 (0.90)	1 (0.43)	1 (0.23)	
Depression score, mean ± SD	8.37 ± 6.75	8.37 ± 6.77	5.11 ± 7.18	9.37 ± 5.77	7.72 ± 6.07	7.84 ± 5.72	<0.001
<16	50,121 (89.28)	48,278 (89.20)	222 (93.67)	1009 (90.66)	209 (89.32)	403 (93.50)	0.004
≥16	6016 (10.72)	5844 (10.80)	15 (6.33)	104 (9.34)	25 (10.68)	28 (6.50)	

Values are presented as mean ± standard deviation or number (%). Group differences were assessed using analysis of variance (ANOVA) for continuous variables and Pearson’s chi-square test for categorical variables. Other staff include clinical laboratory technologists, rehabilitation therapists, pharmacists, radiologic technologists, and other allied health professionals. Work schedule categories were defined as follows: Three-shift = rotating day/evening/night schedule; Two-shift = rotating long-day and night schedule; Every-other-day shift = 24 h duty performed every other day; Fixed night shift = permanent night duty without rotation; Other = work schedules not classified above. Table 1 includes key baseline characteristics; additional variables and full descriptive statistics are available in Table S1.

**Table 2:** Changes in Mental Health Questionnaire Scores Over Time by Occupation.

	Pre-COVID (Reference)	COVID β (95% CI)	*p*-Value	Post-COVID β (95% CI)	*p*-Value	Medical–Government Conflict β (95% CI)	*p*-Value
Stress							
Non-healthcare worker	reference	0.03 (−0.03, 0.09)	0.382	**0.26 (0.19, 0.33)**	**<0.001**	**0.20 (0.13, 0.26)**	**<0.001**
Physician	reference	0.62 (−0.07, 1.30)	0.076	**1.44 (0.64, 2.23)**	**<0.001**	**1.17 (0.34, 2.01)**	**0.006**
Nurse	reference	−0.52 (−1.07, 0.04)	0.068	−0.30 (−0.91, 0.30)	0.331	**−0.91 (−1.49, −0.33)**	**0.002**
Office worker	reference	0.42 (−0.44, 1.27)	0.342	0.82 (−0.14, 1.78)	0.094	−0.16 (−1.10, 0.77)	0.737
Others	reference	−0.56 (−1.13, 0.02)	0.057	**−0.71 (−1.36, −0.06)**	**0.031**	**−1.09 (−1.72, −0.46)**	**0.001**
Anxiety							
Non-healthcare worker	reference	**−0.58 (−0.68, −0.48)**	**<0.001**	**−0.26 (−0.37, −0.14)**	**<0.001**	**−0.28 (−0.40, −0.17)**	**<0.001**
Physician	reference	0.68 (−0.09, 1.44)	0.084	**1.25 (0.28, 2.22)**	**0.012**	**1.36 (0.28, 2.43)**	**0.013**
Nurse	reference	−0.10 (−0.82, 0.61)	0.774	−0.19 (−1.01, 0.63)	0.649	−0.564 (−1.36, 0.28)	0.197
Office worker	reference	0.28 (−0.85, 1.40)	0.629	1.00 (−0.35, 2.36)	0.147	1.15 (−0.24, 2.54)	0.104
Others	reference	**−0.76 (−1.51, −0.01)**	**0.047**	**−1.26 (−2.17, −0.35)**	**0.006**	**−0.94 (−1.88, −0.004)**	**0.049**
Depression							
Non-healthcare worker	reference	**0.79 (0.72, 0.86)**	**<0.001**	**0.91 (0.82, 0.99)**	**<0.001**	**0.87 (0.79, 0.95)**	**<0.001**
Physician	reference	0.11 (−0.44, 0.66)	0.705	0.50 (−0.19, 1.19)	0.152	0.43 (−0.33, 1.18)	0.269
Nurse	reference	0.15 (−0.45, 0.74)	0.626	0.48 (−0.16, 1.12)	0.142	0.20 (−0.41, 0.81)	0.515
Office worker	reference	0.83 (−0.18, 1.84)	0.107	**1.66 (0.56, 2.77)**	**0.003**	**1.74 (0.67, 2.81)**	**0.001**
Others	reference	−0.03 (−0.52, 0.50)	0.916	0.31 (−0.26, 0.89)	0.288	0.55 (−0.02, 1.13)	0.061

Values are presented as estimated mean differences (β) with 95% confidence intervals (CI) from linear mixed-effects models. Pre-COVID period was used as the reference category. Models were adjusted for age, sex, education level, and marital status. Bold indicates *p* < 0.05. Detailed descriptive statistics for each mental health outcome by period and occupation are provided in Table S2.

**Table 3:** Changes in Mental Health Questionnaire Scores Over Time by Length of Service in Physicians.

	Pre-COVID (Reference)	COVID β (95% CI)	*p*-Value	Post-COVID β (95% CI)	*p*-Value	Medical–Government Conflict β (95% CI)	*p*-Value
Stress							
<5 yr	reference	1.36 (−0.48, 3.19)	0.147	**3.25 (1.19, 5.31)**	**0.002**	**2.62 (0.46, 4.78)**	**0.018**
5–14 yr	reference	**1.03 (0.12, 1.95)**	**0.027**	**1.41 (0.31, 2.51)**	**0.012**	**1.49 (0.33, 2.64)**	**0.012**
≧15 yr	reference	1.08 (−0.45, 2.62)	0.167	2.12 (−0.07, 4.31)	0.058	2.41 (−0.15, 4.97)	0.065
Anxiety							
<5 yr	reference	**3.08 (0.80, 5.36)**	**0.008**	**4.35 (1.72, 6.97)**	**0.001**	**4.03 (1.21, 6.84)**	**0.005**
5–14 yr	reference	0.50 (−0.64, 1.64)	0.387	0.72 (−0.81, 2.26)	0.35	1.22 (−0.50, 2.94)	0.165
≧15 yr	reference	0.76 (−0.81, 2.32)	0.343	1.48 (−1.33, 4.29)	0.301	1.86 (−1.58, 5.30)	0.290
Depression							
<5 yr	reference	−0.53 (−2.35, 1.28)	0.563	1.03 (−1.02, 3.08)	0.325	0.72 (−1.45, 2.89)	0.517
5–14 yr	reference	0.56 (−0.17, 1.28)	0.131	0.18 (−0.78, 1.14)	0.709	0.35 (−0.72, 1.41)	0.524
≧15 yr	reference	0.56 (−0.66, 1.78)	0.366	1.03 (−1.20, 3.26)	0.367	1.06 (−1.68, 3.80)	0.449

Values are presented as estimated mean differences (β) with 95% confidence intervals (CI) from linear mixed-effects models. Pre-COVID period was used as the reference category. Models were adjusted for age, sex, education level, and marital status. Bold indicates *p* < 0.05. Detailed descriptive statistics for each mental health outcome by period and occupation are provided in Table S3.

## Data Availability

The data presented in this study are available on request from the corresponding author. The data are not publicly available due to privacy and ethical restrictions.

## Supplementary Materials

The following supporting information can be downloaded at: https://www.mdpi.com/article/10.3390/jcm14238580/s1, Table S1. Detailed baseline characteristics of the study population. Table S2. Changes in Mental Health Questionnaire Scores Over Time by Occupation. Table S3. Changes in Mental Health Questionnaire Scores Over Time by Length of Service in Physicians. Table S4-1. Longitudinal Changes in the Risk of Depression by Occupation. Table S4-2. Longitudinal Changes in the Risk of Moderate to Severe Anxiety by Occupation.

## Abbreviations

The following abbreviations are used in this manuscript:

COVID	Coronavirus Disease 2019
KNHANES	Korea National Health and Nutrition Examination Survey
CES-D	Center for Epidemiologic Studies Depression Scale
CUXOS	Clinically Useful Anxiety Outcome Scale
BMI	Body Mass Index
MERS	Middle East Respiratory Syndrome
