# Supplementary material for: Impact of a Medical–Government Conflict on Healthcare Workers’ Mental Health in a Single Tertiary Hospital
Source: J Clin Med. 2025 Dec 3;14(23):8580. doi: 10.3390/jcm14238580 (PMC12693315; doi:10.3390/jcm14238580)
Supplement: Supplementary file 1 [file jcm-14-08580-s001.zip › Table S1.pdf]

Table S1. Detailed baseline characteristics of the study population

	Total (N=56,137)	Non-healthcare worker (N=54,122)	Physician (N=237)	Nurse (N=1,113)	Office worker (N=234)	Others (N=431)	*p*-value
Sex							<0.001
Male	41,407 (73.76)	40,859 (75.49)	166 (70.04)	75 (6.74)	88 (37.61)	219 (50.81)	
Female	14,730 (26.24)	13,263 (24.51)	71 (29.96)	1,038 (93.26)	146 (62.39)	212 (49.19)	
Age	40.04 ± 12.36	40.21 ± 12.43	42.97 ± 8.87	33.18 ± 8.22	38.02 ± 8.77	36.66 ± 8.86	<0.001
20–29	13,344 (23.77)	12,683 (23.43)	3 (1.27)	494 (44.38)	43 (18.38)	121 (28.07)	<0.001
30–39	18,967 (33.79)	18,180 (33.59)	108 (45.57)	406 (36.48)	103 (44.02)	170 (39.44)	
40–49	11,459 (20.41)	11,086 (20.48)	78 (32.91)	144 (12.94)	60 (25.64)	91 (21.11)	
50–59	8,144 (14.51)	7,969 (14.72)	35 (14.77)	68 (6.11)	24 (10.26)	48 (11.14)	
≧60	4,223 (7.52)	4,204 (7.77)	13 (5.49)	1 (0.09)	4 (1.71)	1 (0.23)	
Length of service	10.34 ± 8.44	10.54 ± 8.43	4.84 ± 6.38	7.64 ± 7.93	11.70 ± 9.53	9.28 ± 8.64	<0.001
<5yr	7,387 (13.16)	6,392 (11.81)	164 (69.20)	588 (52.83)	76 (32.48)	167 (38.75)	<0.001
5-14yr	9,942 (17.71)	9,285 (17.16)	49 (20.68)	359 (32.26)	83 (35.47)	166 (38.52)	
≧15yr	6,366 (11.34)	6,003 (11.09)	24 (10.13)	166 (14.91)	75 (32.05)	98 (22.74)	
Education level							<0.001
High school or below	16,257 (28.96)	16,059 (29.67)	0 (0.00)	100 (8.98)	60 (25.64)	38 (8.82)	
College	21,247 (37.85)	19,700 (36.40)	76 (32.07)	950 (85.35)	155 (66.24)	366 (84.92)	
Graduate school or higher	2,522 (4.49)	2,252 (4.16)	161 (67.93)	63 (5.66)	19 (8.12)	27 (6.26)	
Work schedule							<0.001
Three-shifts	4,921 (8.77)	4,296 (7.94)	1 (0.42)	584 (52.47)	8 (3.42)	32 (7.42)	
Two-shifts	6,912 (12.31)	6,848 (12.65)	3 (1.27)	27 (2.43)	1 (0.43)	33 (7.66)	
Every-other-day shift	52 (0.09)	17 (0.03)	2 (0.84)	2 (0.18)	1 (0.43)	30 (6.96)	
Fixed night shift	57 (0.10)	32 (0.06)	8 (3.38)	5 (0.45)	0 (0.00)	12 (2.78)	
Other	752 (1.34)	540 (1.00)	77 (32.49)	64 (5.75)	2 (0.85)	69 (16.01)	
Marital status							<0.001
Never married	9,782 (17.43)	8,787 (16.24)	49 (20.68)	663 (59.57)	93 (39.74)	190 (44.08)	
Married	28,739 (51.19)	27,757 (51.29)	185 (78.06)	428 (38.45)	138 (58.97)	231 (53.60)	
Separated	105 (0.19)	103 (0.19)	0 (0.00)	1 (0.09)	0 (0.00)	1 (0.23)	
Divorced	997 (1.78)	970 (1.79)	2 (0.84)	16 (1.44)	2 (0.85)	7 (1.62)	
Widowed	575 (1.02)	566 (1.05)	1 (0.42)	5 (0.45)	1 (0.43)	2 (0.46)	
Mental health diagnosis							<0.001
Yes	2,541 (4.53)	2,519 (4.65)	6 (2.53)	7 (0.63)	5 (2.14)	4 (0.93)	
No	53,596 (95.47)	51,603 (95.35)	231 (97.47)	1,106 (99.37)	229 (97.86)	427 (99.07)	
BMI	24.40 ± 3.59	24.45 ± 3.57	24.62 ± 3.99	22.53 ± 3.64	23.20 ± 3.61	23.66 ± 3.82	<0.001
<25	34,141 (60.82)	32,658 (60.34)	135 (56.96)	883 (79.34)	175 (74.79)	290 (67.29)	<0.001
≧25	21,984 (39.16)	21,456 (39.64)	99 (41.77)	230 (20.66)	58 (24.79)	141 (32.71)	
Smoking							<0.001
Non-smoker	28,071 (50.00)	26,344 (48.68)	161 (67.93)	1,074 (96.50)	188 (80.34)	304 (70.53)	
Ex-smoker	11,411 (20.33)	11,265 (20.81)	39 (16.46)	20 (1.80)	21 (8.97)	66 (15.31)	
Current smoker	15,971 (28.45)	15,831 (29.25)	35 (14.77)	19 (1.71)	25 (10.68)	61 (14.15)	
Alcohol							<0.001
<1 time per week	8,853 (15.77)	8,268 (15.28)	54 (22.78)	360 (32.35)	51 (21.79)	120 (27.84)	
1–2 times per week	29,521 (52.59)	28,543 (52.74)	129 (54.43)	509 (45.73)	129 (55.13)	211 (48.96)	
3–4 times per week	6,538 (11.65)	6,422 (11.87)	17 (7.17)	50 (4.49)	16 (6.84)	33 (7.66)	
5–7 times per week	1,153 (2.05)	1,140 (2.11)	5 (2.11)	3 (0.27)	2 (0.85)	3 (0.70)	

Physical activity							<0.001
Yes	21,490 (38.28)	20,785 (38.40)	56 (23.63)	398 (35.76)	81 (34.62)	170 (39.44)	
No	34,379 (61.24)	33,069 (61.10)	181 (76.37)	715 (64.24)	153 (65.38)	261 (60.56)	
Stress score	15.25 ± 6.50	15.23 ± 6.51	15.03 ± 5.86	15.95 ± 6.36	16.26 ± 6.48	14.90 ± 5.73	<0.001
Anxiety score	12.37 ± 12.39	12.38 ± 12.42	10.89 ± 10.76	12.22 ± 11.64	14.34 ± 12.51	10.73 ± 11.00	0.002
0–25	47,213 (84.10)	45,478 (84.03)	220 (92.83)	949 (85.27)	186 (79.49)	380 (88.17)	0.001
26–50	8,483 (15.11)	8,217 (15.18)	15 (6.33)	154 (13.84)	47 (20.09)	50 (11.60)	
51–80	441 (0.79)	427 (0.79)	2 (0.84)	10 (0.90)	1 (0.43)	1 (0.23)	
Depression score	8.37 ± 6.75	8.37 ± 6.77	5.11 ± 7.18	9.37 ± 5.77	7.72 ± 6.07	7.84 ± 5.72	<0.001
<16	50,121 (89.28)	48,278 (89.20)	222 (93.67)	1,009 (90.66)	209 (89.32)	403 (93.50)	0.004
≧16	6,016 (10.72)	5,844 (10.80)	15 (6.33)	104 (9.34)	25 (10.68)	28 (6.50)	

Values are presented as mean ± standard deviation or number (%). Group differences were assessed using analysis of variance (ANOVA) for continuous variables and Pearson's chi-square test for categorical variables.
