# Supplementary material for: Impact of a Medical–Government Conflict on Healthcare Workers’ Mental Health in a Single Tertiary Hospital
Source: J Clin Med. 2025 Dec 3;14(23):8580. doi: 10.3390/jcm14238580 (PMC12693315; doi:10.3390/jcm14238580)
Supplement: Supplementary file 1 [file jcm-14-08580-s001.zip › Table S4.pdf]

Table S4-1. Longitudinal Changes in the Risk of Depression by Occupation

| | Pre-COVID (reference) | COVID OR (95% CI) | *p*-value | Post-COVID OR (95% CI) | *p*-value | Medical—government conflict OR (95% CI) | *p*-value |
|---|---|---|---|---|---|---|---|
| Non-healthcare worker | reference | 1.00 (0.96, 1.04) | 0.911 | **1.15 (1.10, 1.20)** | **<0.001** | **1.14 (1.09, 1.19)** | **<0.001** |
| **Doctor** | reference | 1.14 (0.63, 2.06) | 0.663 | 1.43 (0.74, 2.73) | 0.285 | 1.27 (0.64, 2.52) | 0.487 |
| **Nurse** | reference | 1.15 (0.78, 1.69) | 0.482 | 1.22 (0.81, 1.83) | 0.347 | 0.86 (0.58, 1.28) | 0.470 |
| **Office worker** | reference | 1.01 (0.64, 1.59) | 0.970 | 1.14 (0.69, 1.88) | 0.619 | 0.50 (0.92, 2.45) | 0.108 |
| **Others** | reference | 0.74 (0.50, 1.10) | 0.139 | 1.15 (0.75, 1.75) | 0.527 | 0.84 (0.55, 1.29) | 0.433 |

A CES-D score of 16 or higher was considered indicative of a potential diagnosis of depression. *P*-values were calculated using Generalized Estimating Equations (GEE). Adjusted for age, sex, education level and marital status. Bold indicates $p < 0.05$. OR, Odds Ratio; CI, Confidence Interval.

Table S4-2. Longitudinal Changes in the Risk of Moderate to Severe Anxiety by Occupation

| | Pre-COVID (reference) | COVID OR (95% CI) | *p*-value | Post-COVID OR (95% CI) | *p*-value | Medical—government conflict OR (95% CI) | *p*-value |
|---|---|---|---|---|---|---|---|
| Non-healthcare worker | reference | **0.89 (0.86, 0.92)** | **<0.001** | 1.03 (0.99, 1.06) | 0.153 | **1.04 (1.00, 1.07)** | **0.048** |
| **Doctor** | reference | 1.84 (0.49, 6.94) | 0.371 | 1.79 (0.44, 7.25) | 0.412 | 1.96 (0.50, 7.68) | 0.332 |
| **Nurse** | reference | 1.11 (0.63, 1.96) | 0.728 | 1.22 (0.68, 2.16) | 0.508 | 0.75 (0.43, 1.30) | 0.310 |
| **Office worker** | reference | 1.31 (0.88, 1.94) | 0.185 | 1.51 (0.97, 2.34) | 0.069 | 1.31 (0.84, 2.04) | 0.236 |
| **Others** | reference | 1.02 (0.70, 1.48) | 0.925 | 1.07 (0.70, 1.64) | 0.747 | 1.20 (0.78, 1.83) | 0.401 |

The outcome was defined as moderate-to-severe anxiety (CUXOS score ≥ 26). Scores were categorized as mild (0–25), moderate (26–50), and severe (51–80). *P*-values were calculated using Generalized Estimating Equations (GEE). Adjusted for age, sex, education level and marital status. Bold indicates $p < 0.05$. OR, Odds Ratio; CI, Confidence Interval.
