# Supplementary material for: Impact of a Medical–Government Conflict on Healthcare Workers’ Mental Health in a Single Tertiary Hospital
Source: J Clin Med. 2025 Dec 3;14(23):8580. doi: 10.3390/jcm14238580 (PMC12693315; doi:10.3390/jcm14238580)
Supplement: Supplementary file 1 [file jcm-14-08580-s001.zip › Table S3.pdf]

Table S3. Changes in Mental Health Questionnaire Scores Over Time by Length of Service in Physicians

	**Pre-COVID (reference)**	**COVID β (95% CI)**	***p*-value**	**Post-COVID β (95% CI)**	***p*-value**	**Medical—government conflict β (95% CI)**	***p*-value**
Stress							
<5yr	13.42 (11.30, 15.54)	14.77 (13.47, 16.08)	0.147	**16.66 (15.29, 18.04)**	**0.002**	**16.04 (14.68, 17.39)**	**0.018**
5–14 yr	13.21 (12.12, 14.31)	**14.25 (13.26, 15.24)**	**0.027**	**14.63 (13.53, 15.72)**	**0.012**	**14.70 (13.58, 15.82)**	**0.012**
≧15yr	15.67 (13.17, 18.17)	16.75 (14.55, 18.96)	0.167	17.79 (15.45, 20.12)	0.058	18.08 (15.59, 20.56)	0.065
Anxiety							
<5yr	7.84 (4.78, 10.90)	**10.92 (8.74, 13.10)**	**0.008**	**12.19 (9.96, 14.41)**	**0.001**	**11.87 (9.64, 14.09)**	**0.005**
5–14 yr	9.63 (7.61, 11.66)	10.13 (8.26, 12.01)	0.387	10.36 (8.37, 12.34)	0.356	10.85 (8.80, 12.91)	0.165
≧15yr	12.34 (8.21, 16.47)	13.10 (9.35, 16.84)	0.343	13.82 (9.97, 17.67)	0.301	14.20 (10.14, 18.26)	0.290
Depression							
<5yr	5.49 (3.28, 7.70)	4.96 (3.51, 6.41)	0.563	6.52 (5.02, 8.03)	0.325	6.21 (4.72, 7.70)	0.517
5–14 yr	3.21 (1.99, 4.42)	3.76 (2.64, 4.89)	0.131	3.39 (2.19, 4.58)	0.709	3.55 (2.32, 4.79)	0.524
≧15yr	6.39 (2.94, 9.84)	6.95 (3.79, 10.11)	0.366	7.42 (4.18, 10.66)	0.367	7.45 (4.05, 10.85)	0.449

Values are presented as mean with 95% confidence intervals (CI) from linear mixed-effects models. Pre-COVID period was used as the reference category. Models were adjusted for age, sex, education level, and marital status. Bold indicates $p < 0.05$.
