# Supplementary material for: Impact of a Medical–Government Conflict on Healthcare Workers’ Mental Health in a Single Tertiary Hospital
Source: J Clin Med. 2025 Dec 3;14(23):8580. doi: 10.3390/jcm14238580 (PMC12693315; doi:10.3390/jcm14238580)
Supplement: Supplementary file 1 [file jcm-14-08580-s001.zip › Table S2.pdf]

Table S2. Changes in Mental Health Questionnaire Scores Over Time by Occupation

	**Pre-COVID (reference)**	**COVID β (95% CI)**	***p*-value**	**Post-COVID β (95% CI)**	***p*-value**	**Medical—government conflict β (95% CI)**	***p*-value**
**Stress**							
Non-healthcare worker	15.20 (15.13, 15.27)	15.23 (15.17, 15.28)	0.382	**15.46 (15.39, 15.52)**	**<0.001**	**15.39 (15.33, 15.46)**	**<0.001**
Physician	14.38 (13.41, 15.35)	15.00 (14.16, 15.83)	0.076	**15.82 (14.95, 16.68)**	**<0.001**	**15.55 (14.69, 16.41)**	**0.006**
Nurse	16.62 (15.96, 17.28)	16.11 (15.54, 16.67)	0.068	16.32 (15.76, 16.89)	0.331	**15.71 (15.30, 16.12)**	**0.002**
Office worker	16.40 (15.27, 17.52)	16.81 (15.79, 17.83)	0.342	17.21 (16.20, 18.22)	0.094	16.24 (15.35, 17.12)	0.737
Others	15.59 (14.83, 16.34)	15.03 (14.34, 15.71)	0.057	**14.87 (14.19, 15.56)**	**0.031**	**14.49 (13.91, 15.08)**	**0.001**
**Anxiety**							
Non-healthcare worker	13.07 (12.93, 13.20)	**12.48 (12.38, 12.59)**	**<0.001**	**12.81 (12.68, 12.94)**	**<0.001**	**12.78 (12.66, 12.90)**	**<0.001**
Physician	10.29 (8.72, 11.87)	10.97 (9.54, 12.39)	0.084	**11.54 (10.12, 12.97)**	**0.012**	**11.65 (10.23, 13.07)**	**0.013**
Nurse	13.23 (12.14, 14.31)	13.12 (12.18, 14.06)	0.774	13.04 (12.14, 13.94)	0.649	12.68 (11.95, 13.42)	0.197
Office worker	14.59 (12.52, 16.67)	14.87 (12.98, 16.76)	0.629	15.59 (13.78, 17.41)	0.147	15.74 (14.06, 17.43)	0.104
Others	12.27 (10.89, 13.65)	**11.51 (10.26, 12.76)**	**0.047**	**11.01 (9.81, 12.21)**	**0.006**	**11.33 (10.22, 12.43)**	**0.049**
**Depression**							
Non-healthcare worker	7.36 (7.28, 7.44)	**8.15 (8.09, 8.21)**	**<0.001**	**8.27 (8.19, 8.34)**	**<0.001**	**8.23 (8.16, 8.30)**	**<0.001**
Physician	4.89 (3.81, 5.97)	4.99 (4.02, 5.97)	0.705	5.39 (4.42, 6.36)	0.152	5.31 (4.34, 6.29)	0.269
Nurse	8.49 (7.84, 9.13)	8.63 (8.08, 9.19)	0.626	8.97 (8.41, 9.53)	0.142	8.69 (8.30, 9.07)	0.515
Office worker	6.22 (5.01, 7.44)	7.05 (5.96, 8.15)	0.107	**7.89 (6.79, 8.98)**	**0.003**	**7.96 (7.02, 8.91)**	**0.001**
Others	6.80 (6.05, 7.55)	6.78 96.10, 7.45)	0.916	7.11 (6.45, 7.78)	0.288	7.35 (6.76, 7.94)	0.061

Values are presented as mean with 95% confidence intervals (CI) from linear mixed-effects models. Pre-COVID period was used as the reference category. Models were adjusted for age, sex, education level, and marital status. Bold indicates $p < 0.05$.
